# An adaptable research platform for ex vivo normothermic machine perfusion of the liver

**DOI:** 10.1007/s11548-023-02903-4

**Published:** 2023-04-24

**Authors:** M. Magbagbeola, Z. L. Rai, K. Doyle, L. Lindenroth, G. Dwyer, A. Gander, A. Stilli, B. R. Davidson, D. Stoyanov

**Affiliations:** 1 Wellcome/EPSRC Centre for Interventional and Surgical Sciences (WEISS), University College London, London, UK; 2 Centre for Surgical Innovation, Organ Repair and Transplantation (CSIORT), UCL, London, UK; 3 Royal Free Hospital NHS Trust, London, UK; 4 Department of Surgical and Interventional Engineering, School of Biomedical Engineering and Imaging Sciences, King’s College London, London, UK

**Keywords:** Liver preservation, Systems design, Machine perfusion

## Abstract

****Purpose**:**

This paper presents an assessment of a low-cost organ perfusion machine designed for use in research settings. The machine is modular and versatile in nature, built on a robotic operating system (ROS2) pipeline allowing for the addition of specific sensors for different research applications. Here we present the system and the development stages to achieve viability of the perfused organ.

****Methods**:**

The machine’s perfusion efficacy was assessed by monitoring the distribution of perfusate in livers using methylene blue dye. Functionality was evaluated by measuring bile production after 90 min of normothermic perfusion, while viability was examined using aspartate transaminase assays to monitor cell damage throughout the perfusion. Additionally, the output of the pressure, flow, temperature, and oxygen sensors was monitored and recorded to track the health of the organ during perfusion and assess the system’s capability of maintaining the quality of data over time.

****Results**:**

The results show the system is capable of successfully perfusing porcine livers for up to three hours. Functionality and viability assessments show no deterioration of liver cells once normothermic perfusion had occurred and bile production was within normal limits of approximately 26 ml in 90 min showing viability.

****Conclusion**:**

The developed low-cost perfusion system presented here has been shown to keep porcine livers viable and functional ex vivo. Additionally, the system is capable of easily incorporating several sensors into its framework and simultaneously monitor and record them during perfusion. The work promotes further exploration of the system in different research domains.

## Introduction

Advancements in surgical techniques and pharmacological interventions have improved the efficacy of organ transplantation in recent years [1]. However, the demand for organs is increasing with no apparent change in organ donation rates. One solution that is currently being evaluated is machine perfusion (MP). Ex vivo organ perfusion is a technique intended to improve the viability of organs to allow the use of donor organs that would previously have been discarded [2]. It has also allowed organ transplant to be timed to fit with other clinical activities, making it a beneficial tool for future clinical use.

From a systems perspective, there have been many perfusion machines delivered to the market and used in clinical settings that successfully perfuse organs such as livers for up to 24 h without compromising patient outcomes [3]. However, these commercial machines are not widely used within the research domain, due in part to their high costs, lack of adaptability, and requirement of a specialized team to operate. For example, the OrganOx Metra costs approximately £30,000 per annum to lease with over £6,000 in disposable costs [4] and does not allow for post-analysis of data such as pressure, flow, oxygen concentration, or temperature. Additionally, access to the system is often restricted preventing researchers from modifying the system to suit their needs.

In the research domain, more specialized perfusion systems have been developed for different organs with some functionality of peripheral monitoring [5, 6]. Eshmuminov et al [7] developed a perfusion machine with the aim of preserving pig and human livers for up to 1 week. With their system, they were able to monitor parameters such as pressure, flow, glucose, and oxygen concentration and to provide real-time control. Other researchers have begun to explore closed-loop controllers to control hemodynamic parameters such as pressure or flow rate for other organs in perfusion machines to varying degrees of success [8–11]. Though these systems offer more functionality than their commercial counterparts, they offer little adaptability to different experimental needs and are often specialized to the targeted organ. Additionally, these MP circuits have been developed for maintaining and improving organ viability and function for transplantation, as such, several key parameters not directly related to viability go unobserved and unmodulated which may be of great interest to researchers.

The customized ex vivo normothermic machine perfusion (NMP) circuit presented here has been designed and developed specifically with the aim of being versatile and modular to suit a heterogeneous mix of disciplines in research. The machine has been built using low-cost, replaceable, and interchangeable sensors and a microcomputer for centralized simultaneous real-time control, monitoring, and recording of all added sensors and actuators. It has a total parts cost of £11,000 with approximately £30 per perfusion in disposable costs, considerably cheaper than commercial machines. The modular nature of the machine allows researchers to add or remove real-time monitoring parameters that are of most interest and applicability such as additional pressure, flow, or glucose sensors while remaining independent to each other. This paper presents the system and its application toward the perfusion of fresh porcine livers to demonstrate its efficacy at attaining perfusion successfully.

**Fig. 1:**
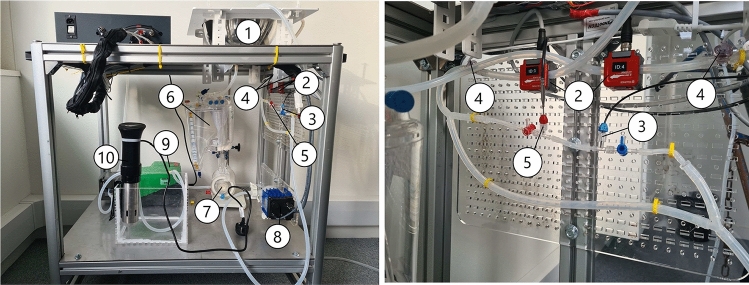
Figure shows the organ perfusion system platform. (1) Organ chamber, (2) flow sensors, (3) temperature sensor, (4) pressure sensor, (5) oxygen sensor, (6) reservoir, (7) oxygenator, (8) centrifugal pump, (9) peristaltic pump, (10), heat exchanger. The figure on the right shows an enlarged image of the sensor configuration found in the figure on the left

## Materials and methods

### System overview

The perfusion machine, presented in Fig. 1, consists of low-cost materials that are easily replaceable and adaptable. An organ chamber, designed to hold the liver or other organs, is located on the topside of a movable metallic cart and is connected to a reservoir by means of silicone tubing. The reservoir with attached oxygenator (Terumo, CAPIOX FX) is modified for multi-use by gaining access to the inner filters allowing for their replacement and deep cleaning. It has a capacity to hold up to 4 L of perfusate. Oxygen is provided by a standard 2-L pressurized cylinder containing medical grade oxygen (BOC, Oxygen 1-E) the supply of which is manually adjusted via a regulator.

**Fig. 2:**
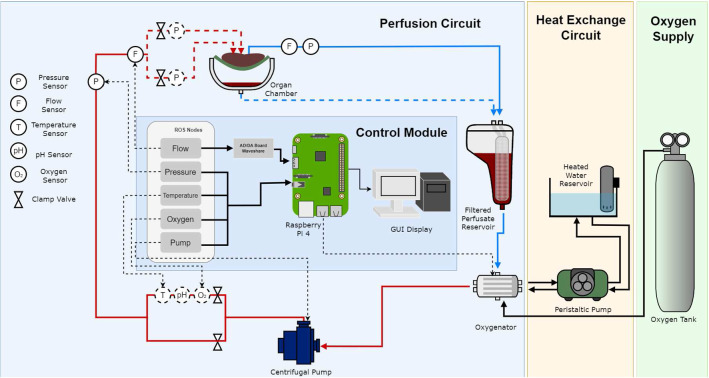
Schematic overview of the proposed perfusion system. The flow perfusion system circuit diagram is indicated by the red and blue lines corresponding to oxygenated and deoxygenated blood, respectively. Various sensors are placed on the input stream into the organ as well as a single pressure and flow sensor on the output stream leading back to the reservoir. The Raspberry Pi acts as the control module for all sensing on the system controlled by ROS2 topics. A GUI is also available for direct control and monitoring of all sensors. The dotted circles indicate sensors added at a later developmental stage of the design

All peripherals are controlled via a Raspberry pi Model 4 single board computer and interfaced using a combination of MODBUS, RS-232 and RS-485 communication protocols. An analog to digital converter board was used to interface the Raspberry pi and the flow sensors. The software architecture has been all implemented in a robotic operating system (ROS2) framework. Using this framework, the peripheral sensors can be modularized into nodes that communicate with each other using a publisher/subscriber protocol despite the differences in underlying software. The Raspberry pi monitors all sensor data in real time and records the output for further analysis. A schematic overview of the perfusion circuit is shown in Fig. 2. A single centrifugal pump (PuraLev 1200 MU) was used to pump oxygenated blood to the portal vein (PV) and hepatic artery (HA). Two pressure (PendoTech, Press-n-075) and flow (Sonotec sonoglow, co.55/100) sensors were used to monitor both the input and the output pressure and flow rates, while oxygen (PreSens, EOM-(t)-FOM), and temperature (PreSens, Pt100), sensors were used to monitor the input stream only. The tested organ sits in an organ chamber whose output and runoff are collected into a reservoir capable of holding up to 4 L of perfusate where it is then filtered. The oxygenator, connected to the reservoir, re-oxygenated the blood before it was pumped back through the system. Blood was warmed via the heat exchange circuit controlled via a peristaltic pump (Watson-Marlow, 520 DU). Further tests conducted on the system demonstrate its capability of maintaining set sensing measurements such as pressure via the use of a proportional integral derivative (PID) controller [12].

### System evaluation & Organ retrieval

The system was evaluated by exploring the capability of the design in successfully perfusing a porcine liver for up to three hours while simultaneously monitoring and recording sensing data. Specific qualities to assess perfusion were established in three stages: visual evaluation of dispersion of perfusate in the liver, addition of sensors and assessment of quality of data following extended perfusion time, and assessment of viability and functionality of the liver following perfusion. Table 1 shows different stages of the evaluation development process and the sensors incorporated in each stage. All porcine livers were taken immediately following termination from a commercial abattoir and were subjected to the same organ retrieval and cannulation procedure. On receipt of the organ and blood at our dedicated perfusion laboratory, the PV and HA were cannulated using silicone tubing secured with cable ties and served as two dedicated input routes, whereas the hepatic vein (HV) was cannulated and served as the dedicated output route. These vessels were chosen to mirror as much as possible the fluid dynamics in normal physiology.

**Table 1:**
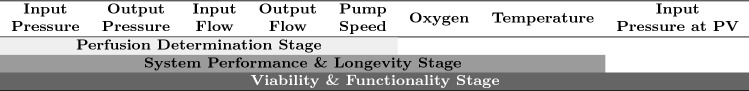
Experimental stages of the study and the sensing modalities added at each stage. Blood is introduced as a perfusate during the system phase, thus requiring the need for oxygen and temperature monitoring to keep the organ viable

#### Phase 1: Assessment of organ perfusion

To determine whether the system as proposed could successfully homogeneously perfuse a porcine liver, methylene blue dye was injected into a mixture of normal saline with a ratio of 1:20 and used as perfusate. Methylene blue dye is used regularly in clinical practice to evaluate leaks following the stapling or anastomosis between hollow viscera. It is specifically used as it is a striking colored blue dye that can be readily and easily visualized intraoperatively. The liver was placed in the organ chamber with input (HA and PV) and output (HV pressure and flow monitored via a graphical user interface). Manual adjustment of the centrifugal pump was established and used to directly control the pressure and flow rate of the solution into the liver by setting the RPM value of the motor and monitoring the response. This was set to perfuse for approximately 45 min at an input pressure of approximately 75 mmHg. This experiment was repeated on three separate occasions and the visual results recorded at the start of the perfusion, halfway through, and at the end for each experiment.

#### Phase 2: System performance & longevity

The purpose of this phase was to determine whether the system could perfuse a liver using whole autologous blood, donated from the pig following cardiac death and how long perfusion could be maintained under these conditions. In all cases, 3 L of blood was used to prime the system and approximately 20,000 IU of heparin was added to the perfusate prior to the experiment. The blood was then warmed via the heat exchanger circuit build into the perfusion system and oxygen provided manually, as shown in Fig. 2. Additional real-time temperature and oxygen sensors were incorporated into the system along with the pressure and flow sensors already present, as described in the previous phase. System performance experiments were conducted on three livers by assessing how well the system could reach and stably maintain target hemodynamic parameters. The target pressure was approximately 75 mmHg and was chosen to closely replicate the normal physiology of input pressure as found in a healthy porcine liver. The additional parameters of temperature and oxygen had target parameters of approximately 29.5$^\circ C$ and 95% concentration, respectively, again chosen to mimic normal physiology. In addition to this, longevity tests were conducted under the same conditions on three additional livers in which the organ was set to maintain perfusion for up to three hours. This was done to determine whether the proposed system could successfully provide normothermic perfusion to a liver for an extended period of time while simultaneously recording and monitoring all sensing data.

#### Phase 3: Viability and functionality test

The objective of phase 3 was to evaluate organ viability and functionality following normothermic perfusion. Viability was based on the adequacy of perfusion along with a measurement of hepatocellular injury by measuring aspartate transaminase (AST) levels in the perfusion circuit. The setup for this phase was identical to phase 2, with the addition of another pressure sensor allowing for individual pressure measurements of the HA and PV. AST was measured using a fluorometric AST assay kit (abcam ab 138878). 10 ml of perfusate was drawn off the perfusion system, via a dedicated port, at baseline (prior to the start of perfusion) and then every 15 min for 90 min. Oxygen and temperature levels remained consistent at target levels for the duration of the experiments. All other sensors remained in the same configuration as previous phases and were continuously monitored and recorded for approximately 90 min.

## Results

### Phase 1 Results

**Fig. 3:**
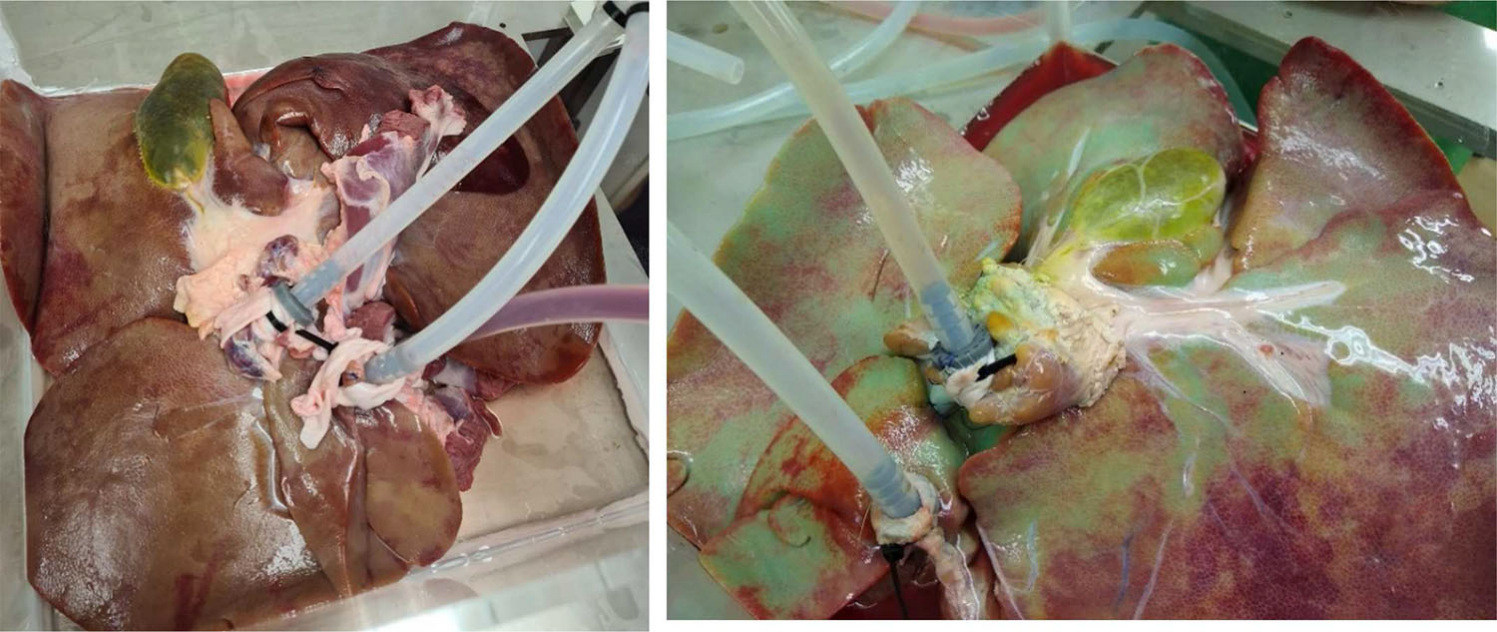
Phase 1 results. Left: before addition of methylene blue; right: dispersion after experiment. The striking color of the blue dye helps identify areas where the system has been able to successfully disperse in the vasculature of the liver

The methylene blue dye was seen to disperse throughout the organ in all three cases examined within 30–50 min of perfusion. Figure 3 shows a perfused liver before and after perfusion (45 min duration) with methylene blue administration. The distinct blue color can be seen in the surrounding vasculature and lobes of the liver. By the end of the perfusion blue shading appears in large portions of the liver, demonstrating that on a macroscopic level; there is good perfusion of the organ using this system.

**Fig. 4:**
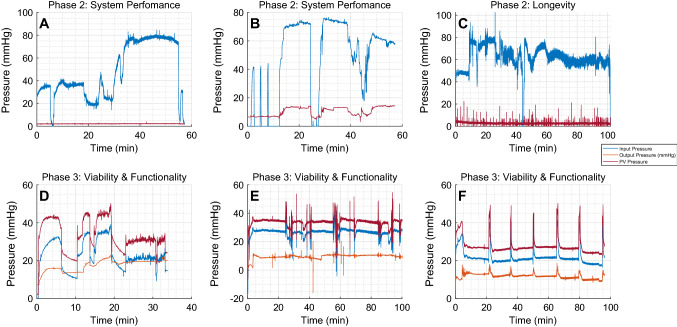
**A**, **B** Perfusion pressure (mmHg) during the system evaluation phase in the presence of donated blood and oxygen. **C** Phase 2 longevity study shows pressure over a longer duration. **D**,**E**,**F** Pressure at HA, PV, and HV during the phase 3 AST study

### Phase 2 System performance & Longevity results

**Fig. 5:**
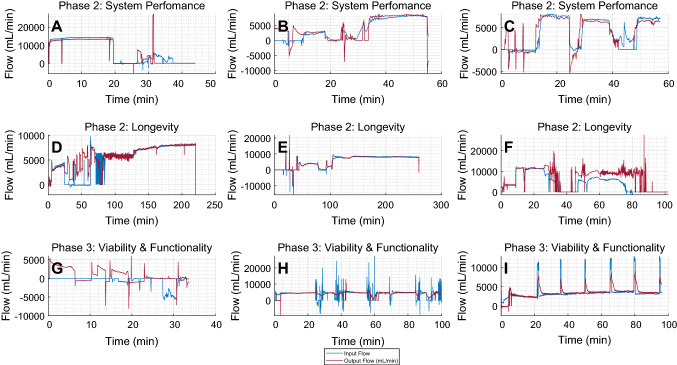
**A**, **B**, **C** Perfusion flow (mL/min) during the system evaluation stage of the study. **D**, **E**, **F** Data from the longevity study. **E** Longest experiment reaching a peak of up to 260 min. **G**, **H**, **I** Input and output flow during the AST measurements. **B**, **C**, **F**, **G**, **H**, and **I** correspond to the pressure data **A**, **B**, **C**, **D**, **E**, and **F**, respectively, in Fig. 4

Figures 4A-C and 5A-F showcase the pressure and flow data recorded from this phase. In all of the system performance experiments, the target pressure of approximately 75 mmHg was reached, as shown in Fig. 4 A,B. In several instances the pressure and flow readings dropped to zero; this was in line with the pump rpm readings and due to stoppage of blood flow to suture lacerations in the organ. Despite these fluctuations once the target parameter was reached, it remained stable over time. The blue line indicates input pressure through both the HA and PV, while the red indicates output pressure at the HV. The output readings in Fig. 4 A and C remain at zero throughout the experiment due to a faulty sensor. Figure 4 B shows relative similarities in pressure trend over time but at a reduced pressure. This relates to input and output flow data seen in Fig. 5A–F. These figures show that input and output flow remains at parity for the majority of the experiment. Though fluctuations can be seen throughout the results, in line with pump and pressure readings. Once target pressure is reached both pressure and flow readings remain stable achieving NMP for up to an hour consistently.

The longevity results from the data gathered from the system show accurate real-time monitoring of several sensors and actuators for up to three hours. However, pressure measurements were not available for 2 experiments and showed noise in the data recorded from the sensor (Fig. 4C). Input and output flow can be seen to stably show perfusion for up to three hours as shown in Fig. 5 D and E. Initial flow measurements fluctuated but typically reached between 5 L/min and 10 L/min. Pump speed was relatively consistent throughout both experiments and was maintained at approximately 3000 RPM. A target temperature of approximately 29.5$^\circ C$ was set, and the recorded data show the temperature settled at the target temperature after approximately 100 mins. Oxygen concentration was manually adjusted to maintain 95% concentration.

### Phase 3 Results

**Fig. 6:**
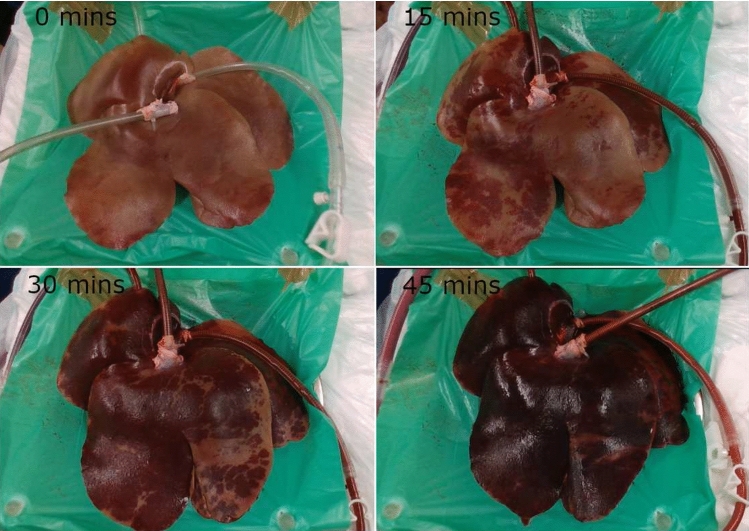
Porcine liver connected undergoing NMP for approximately 60 min. It is evident from visual inspection how well the organ reperfuses over the 60-minute duration. Some areas of the liver did not obtain the typical discoloration normally seen from the introduction of oxygenated blood. This is potentially due to clots in the microvasculature preventing successful perfusion. Overall, the system is capable of successfully perfusing a porcine liver

The points at which samples of AST were taken can clearly be seen in the pressure and flow diagrams of the functionality section in Fig. 4 (D, E, and F) and Fig. 5 (G, H, and I). From a systems perspective the sensing data were more stable and consistent compared to the previous phases for the duration of the experiments and showed little to no variation outside of when the AST samples were extracted. The addition of the PV pressure sensor can also be seen and follows the same patterns of behavior as the other sensors, in line with the pump readings. Perfusion was reached soon after the start of the experiment and remained consistent for up to 100 min of recorded data (Fig. 6).

**Fig. 7:**
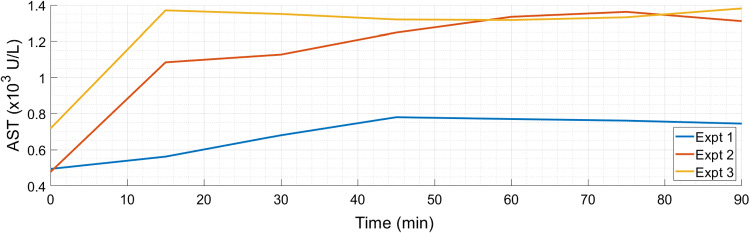
Injury markers of AST shown in three separate experiments during phase 3 of the study and slight initial rise in AST levels followed by a plateau in each case. This indicates that there is no further damage to the liver during the experiments

Figure 7 demonstrates the levels of AST in the perfusate across the duration of 3 perfusion experiments. Following an initial increase, the AST level in the perfusate remained constant throughout the perfusion period. In all normothermic perfusion experiments conducted, small quantities of bile were produced, as shown in Table 2. On average over the three experiments, the liver produced 26 ml of bile in 90 min.

**Table 2:** AST marker levels measured every 15 min for 90 min as well as corresponding bile production output. The averaged bile output was approximately 26 ml in 90 min which is within the realm of normal bile production

	Experiment	15 min	30 min	45 min	60 min	75 min	90 min	Bile produced (ml)
Expt 1	0.494	0.562	0.681	0.780	0.770	0.761	0.745	28
Expt 2	0.478	1.08	1.13	1.25	1.33	1.36	1.31	26
Expt 3	0.718	1.37	1.35	1.32	1.32	1.33	1.38	24

## Discussion

To our knowledge, this is the first report of a custom-built, low-cost, modular perfusion device built on open-source hardware (Raspberry Pi) and software (ROS2). In the initial experiments, fewer sensors were used to monitor the liver and the perfusate was a simple solution of 0.9% normal saline. Over time, blood as a perfusate and additional sensors of temperature and oxygen were added. These iterations were facilitated by the ROS2 framework which allowed for a modular design to the system as each sensor could be incorporated, scaled, and partitioned without interference from the other systems already in place. This, along with the reconfigurable and removable silicone tubing, allows easy adaptation for specific research needs and sterilization. Current experiments have investigated the versatility of the device by exploring its efficacy in perfusing porcine pancreases. The system has also been used in different scientific domains such as investigating perfusate dispersal in a porcine liver under MRI, perfusion with spectral imaging, and with irreversible electroporation.

Heparin was added to later experiments to prevent formation of clots in the donated blood during transportation as seen in phase 1 experiments. The heparin was able to successfully minimize the likelihood of clot formation to allow for better perfusion. Some of the variability seen in the sensing data is due to the presence of small leaks from lacerations on the organ obtained during organ retrieval and subsequent stoppage of blood flow to suture them close. In some cases, despite suturing, small leaks were still evident. However, it was determined that some of the variability in the sensing data was due to other factors, such as the perfusate being too aerated by the oxygenator, faulty sensors, or electrical interference. Steps were taken to mitigate these factors and subsequent experimental data can be seen to improve in quality. For example, oxygen gas input pressure limited to a maximum blood oxygen saturation of 95% preventing over aeration and the faulty sensors replaced. Pressure differences between the HA, PV, and HV are likely due to blood first collecting in the liver prior to output flow being achieved. This also explains the overall change in size of the liver over the course of the experiment as well as the initial difference in input and output flow. This indicates that once perfusion is achieved stable input and output pressure and flow is maintained.

Bile production is a common indicator of viability within the literature. In normal porcine bile production from healthy livers approximately, 400–600 ml of bile is produced in a 24-hour period [13]. Extrapolating from the production over 90 min we estimate that our system can potentially enable the production of approximately 416 ml of bile in a 24-hour period which is within the threshold of healthy liver bile production, however further experiments will be conducted to validate this. AST is found within healthy hepatocytes cell membrane. Damage results in leakage into the circulation or perfusate and therefore indicates that high perfusate AST levels reflect ongoing hepatocyte injury. The AST results suggest that the overall health of the liver is maintained during the perfusion procedure and that there is no further insult to the liver during perfusion. The initial increase in AST levels seen in all three experiments reflects the hepatic stress caused by reperfusion injury and is well documented in other hepatic perfusion experiments.

In the future, other viability factors such as lactate concentration and a more continuous measurement of pH will be explored. Future exploration will also continue to adapt the device to allow for better monitoring and control of key functional parameters relevant to different organs and disciplines explored.

## Conclusion

In conclusion, we have presented a new organ perfusion machine, built using low-cost components on a ROS2 framework. The work presented here shows systems ability to successfully perfuse several porcine livers while simultaneously recording necessary sensors. The viability and functionality test results indicate that the machine can keep the liver alive and functional for an extended period ex vivo. This works promotes further exploration of the capability of the system and its use in alternative research domains.
